# Survival, CD4 T lymphocyte count recovery and immune reconstitution pattern during the first-line combination antiretroviral therapy in patients with HIV-1 infection in Mongolia

**DOI:** 10.1371/journal.pone.0247929

**Published:** 2021-03-08

**Authors:** Solongo Bayarsaikhan, Davaalkham Jagdagsuren, Batbaatar Gunchin, Tsogtsaikhan Sandag

**Affiliations:** 1 AIDS/ STI Research and Surveillance Division, National Center for Communicable Diseases, MoH, Ulaanbaatar, Mongolia; 2 Department of Immunology, School of Biomedicine, Mongolian National University of Medical Sciences, Ulaanbaatar, Mongolia; Chang Gung Memorial Hospital and Chang Gung University, Taoyuan, Taiwan, TAIWAN

## Abstract

Mongolia has a low incidence of human immunodeficiency virus (HIV) infection, with 281 cases reported at the end of 2019 and an estimated incidence rate of <0.01 cases per 1000 population.

However, no study has analyzed the association between antiretroviral therapy (ART) outcomes and pretreatment characteristics of patients with HIV/acquired immunodeficiency syndrome (AIDS) in Mongolia. This retrospective study aimed to determine the survival, CD4 T cell recovery, and immune reconstitution pattern during ART in HIV patients and to determine baseline patient characteristics associated with ART outcomes. Based on three different World Health Organization (WHO) guidelines, we analyzed the 3-year observation data of 166 patients with HIV/AIDS who received treatment between 2010 and 2017. An increase of >50 cells/μL indicated CD4 T cell count recovery, and a cell count of ≥500 cells/μL in patients with a baseline cell count of <500 cells/μL indicated immune reconstitution. In this study, the 3- and 1-year mortality rates were 5.4% (survival rate: 94.6%) and 3.6%, respectively. A total of 83% of deaths that occurred in the observation time occurred within the first 3 months. The CD4 T cell count recovery rates at 3, 12, and 36 months were 62.7%, 80.7%, and 89.2%, respectively. The CD4 T cell count increased to >500 cells/μL in 95 of 145 (65.5%) patients with a baseline cell count of <500 cells/μL after 36 months of ART. The baseline CD4 T cell count was found to be a sensitive indicator for immune reconstitution. An advanced pretreatment clinical stage of HIV infection (as classified by the WHO classification), a low CD4 T cell count in the peripheral blood, and a high viral load before the initiation of the first-line ART accurately predicted survival, CD4 T cell count recovery, and immune reconstitution in Mongolian patients with HIV/AIDS.

## Introduction

The human immunodeficiency virus (HIV) is a lentivirus a subgroup of retrovirus that causes HIV infection and over time acquired immunodeficiency syndrome (AIDS) [1, 2].

Mongolia is a low incidence country for HIV infection with 281 cases reported in end of 2019 and estimated rate of less than 0.01 cases per 1000 population [3, 4]. Despite the low prevalence of HIV infection in the general population in the past two decades, the number of reported cases is growing exponentially [5]. Control of sexually transmitted infections (STIs) and preventing HIV epidemic has been a priority for the Government of Mongolia for many years and commitment has been shown in many ways. The Law on Prevention of HIV and AIDS amended in 2004 refined a formal structure for combating an HIV and AIDS epidemic and identified the rights and duties of people affected by HIV or AIDS so as to be consistent with international conventions and standards [6].

Since 1992, when was detected first case of HIV infection the HIV spread among population in the country may was sporadic until 2002 with total 3 cases, but period of 2002–2012 characterized with dramatic increase of new cases (from 10 new HIV cases in 2002 to 90 in 2012) followed with gradual decrease (30 new cases in 2017) [5].

Highly active antiretroviral therapy (HAART) or standard antiretroviral therapy (ART) consists of the combination of at least three antiretroviral (ARV) drugs to maximally suppress the HIV virus and stop the progression of HIV disease. Huge reductions have been seen in rates of death and suffering when use is made of a potent ART regimen, particularly in early stages of the disease [7, 8]. Initiating combination antiretroviral therapy can achieve suppression of viral replication and an increase in CD4+ T-cell counts in the majority of patients [9], resulting in dramatic decreases in morbidity and AIDS-related mortality [10].

Since 1992 quit number of reports focused on epidemiological situation and response activities of HIV/AIDS in Mongolia [11]. Several research works was dedicated to molecular epidemiological and virological aspects of HIV infection in the country [12, 13] but there are no research reports addressed to immune disorder state and immunomonitoring during ART in Mongolian patients with HIV/AIDS. Great number of studies in different countries and regions were focused on association of HIV/AIDS patients’ baseline characteristics before initiation of ART with survival [14–22], immune recovery [18, 22–24] and immune reconstitution during the therapy. But reports analyzed association of ART outcome with pretreatment characteristics of HIV/AIDS patients in Mongolia still did not appeared.

Objective of this retrospective study is to clarify survival, CD4 T cell recovery and immune reconstitution pattern of patients with HIV during their antiretroviral therapy and to establish a baseline patient characteristics associated with the ART outcome.

## Materials and methods

According to Law on prevention of HIV infection and AIDS approved by Mongolian Parliament [25] in 2012 each HIV suspected cases should be informed and sent to the Division of AIDS/STI surveillance, National Center for Communicable Diseases either to confirm or deny diagnosis of HIV infection. And all confirmed HIV/AIDS cases go to the Division of AIDS/STI surveillance for regular observation for each 3 months, which includes physical examination, T cell subset count and viral load testing, and go under initiation of combination antiretroviral therapy.

By the end of 2019 year 257 off total 281 confirmed HIV/AIDS cases in the country since 1992 were treated with one 6 different combination regimens (protocols) of ART.

### Patients

In this retrospective study was analyzed data of 3 year (36 months) observation of 166 HIV/AIDS patients treated from November 2010 to November 2017 according to 3 different guidelines recommended by the World Health Organization (WHO guidelines) [26–28]. Different indications for initiation of the first-line ART according to count of peripheral blood CD4 T lymphocytes were prescribed in these guidelines. In particular, CD4 T lymphocyte count lower than 350 cell/μL was the indication for initiation of the first-line cART in the WHO guideline issued in 2010 (WHO guideline 2010) [26] and this point was risen until 500 cell/μL in WHO guideline 2013 [27]. However WHO guideline 2016 [28] provides the initiation of the ART regardless of the CD4 T cell count immediately after the diagnosis of HIV infection.

Baseline demographic, behavioral and clinical data of enrolled in this study patients were collected before the initiation of ART from their medical records and shown in the Tables 1 and 2.

**Table 1:** Demographic and behavior information of patients received ART, Ulaanbaatar, Mongolia, 2010–2017.

Patient information	Males (n = 130)	Females (n = 36)	Total
Age			
Mean ± SD	31.8 ± 8.4	33.6 ± 10.3	32.2 ± 8.9
Median/variative	32.0/17–59	32.0/18–57	32.00/17–59
Age groups			
<20	13/7.8	1/0.6	14/8.4
20–24	15/9.0	6/3.6	21/12.7
25–29	25/15.1	8/4.8	33/19.9
30–34	25/15.1	8/4.8	33/19.9
35–39	30/18.1	4/2.4	34/20.5
40–44	13/7.8	4/2.4	17/10.2
45–49	6/3.6	2/1.2	8/4.8
50–54	2/1.2	1/0.6	3/1.8
55–59	1/0.6	2/1.2	3/1.8
<40	108/65.1	27/16.3	135/81.3
≥40	22/13.3	9/5.4	31/18.7
Total	130/78.3	36/21.7	166/100.0
Sexual behavior*			
Heterosexual	22/13.2	28/16.9	50/30.1
Homosexual	83/50.0		83/50.0
Bisexual	25/15.1		25/15.1
FSW		8/4.8	8/4.8

Abbreviations: FSW, female sex worker; SD, standard deviation

*Sexual behavior of the patients was defined according to the questionnaire of the sentinel survey (IBBS Questionnaire for female sex workers and men who have sex with men)

**Table 2:** Baseline clinical data of patients received ART, Ulaanbaatar, Mongolia, 2010–2017.

Patient data	Males (n = 146)	Females (n = 34)	Total
Clinical stage (number of patients/percentage)			
Stage I	75/45.2	26/15.7	101/60.8
Stage II	7/4.2	2/1.2	9/5.4
Stage III	16/9.6	2/1.2	18/10.8
Stage IV	32/19.3	6/3.6	38/22.9
CD4 T cell count before initiation of ART (cell/μL)			
Mean ± SD	317.8 ± 154.1	364.5 ± 183.7	327.9 ± 161.6
<200	34/20.5	7/4.2	41/24.7
201–350	41/24.7	9/5.4	50/30.1
351–500	42/25.3	12/7.2	54/32.5
>500	13/7.8	8/4.8	21/12.7
Baseline viral load (copies/ml)* (number of patients/percentage)			
High (>100 000)	37//33.3	8/7.2	45/40.5
Medium (10 001–100 000)	30/27.0	9/8.1	39/35.1
Low (≤ 10 000)	18/16.2	9/8.1	27/24.3
ART regimen (number of patients/percentage)			
1D scheme: (TDF +3TC+EFV)	109/65.7	26/15.7	135/81.3
1F scheme: (TDF+FTC+EFV)	21/12.7	10/6.0	31/18.7
ART guidelines			
WHO 2010	8/4.8	1/0.6	9/5.4
WHO 2013	81/48.8	23/13.9	104/62.7
WHO 2016	41/24.7	12/7.2	53/31.9
Tuberculosis (TB) co-infection^†^ (number of patients/percentage)			
Co-infection	27/16.3	2/1.2	29/17.5
TB diagnosed before ART initiation	9/5.4%	0	9/5.4
TB diagnosed in course of the ART	18/10.8	2/1.2	20/12.0

Abbreviations: 3TC, lamivudine; ART, antiretroviral therapy; EFV, efavirenz; FTC, emtricitabine; SD, standard deviation; TB, tuberculosis; TDF, tenofovir; WHO, World Health Organization

*-baseline viral load was available in 111 patients

^†^-22 of TB cases identified as pulmonary TB, and 7 of TB cases–as non-pulmonary TB

### Ethical statement

Ethical issues of the study and consent form were reviewed and approved in Institutional Review Board of Mongolian National University of Medical Sciences in 22 June 2018. Informed consent in written form was received in 159 patients during their ART visit in AIDS/STI division. Data of 7 patients in which AIDS-related death were occurred before ethical approval were included to the study without written consent. The Mongolian Parliament approved the Law on Prevention from Human immunodeficiency virus infection and acquired immunodeficiency syndrome in 2004, and a new version was approved in 2012. Following these laws, numerous regulations were confirmed in the form of orders from the Minister of Health and internal regulations of organizations. According to these regulations, the personal information of patients is strictly classified in order to avoid any form of discrimination. Researchers, medical personnel are prohibited from copying, photographing the personal information from medical documents of the patient. In particular, patients should not declare the closest family ties, if they do not want to. And we had no information about the next of kin for deceased patients. We stated these difficulties with a written permission from the next of kin to the Institutional Review Board. IRB approved the study design.

Informed consent form were designed according to the WHO Informed Consent Form Template for Clinical Studies [29].

### ART regimen and control

Data for three year observation during first-line ART was available in 166 patients received first-line ART. 1D scheme of ART by combination of tenofovir (TDF), lamivudine (3TC) and efavirenz (EFV) was conducted in 135 patients and 1F scheme of ART by combination of tenofovir (TDF), emtricitabine (FTC) and efavirenz (EFV) were conducted in 31 patients.

The term CD4 T cell count recovery to ART with responders or non-responders has an ambiguous meaning [30]. In this study the occurrence of the CD4 T lymphocyte count recovery or response to ART was considered when cell count became increased at least in 50 cells in 1 microliter (cell/μL) of peripheral blood within first and third year [31]. CD4 T lymphocyte recovery was monitored in 3 points of time (3 months, 12 months and 36 months) after the initiation of ART.

Occurrence of the immune reconstitution in patients with baseline immunosupression (with baseline CD4 T cell count less than 500 cell/μL) was considered when cell count reach the level 500 cell/μL and higher. Immune reconstitution was monitored in time 3 points after initiation of ART, same as in CD4 lymphocyte recovery cases.

### T cell subset and viral load

Count of T cell subsets in peripheral blood (cell/μL) of patients were performed by flow cytometry (BD FACSCount^TM^, Becton Dickinson, USA) using BD FACSCount reagent kit for enumerating absolute counts of CD4, CD8, and CD3 T lymphocytes in unlysed whole blood (Catalog No. 340167) [32].

Viral load (copy/μL) was measured by nucleic acid amplification assay (NucliSENS EasyQ^®^ HIV-1 v2.0, bioMérieux SA). Were used NucliSENS EasyQ^®^ HIV-1 v2.0 kit for NASBA^®^-based amplification and real time detection of HIV-1 RNA [33].

### Statistical analysis

Descriptive and analytical statistics were used to analyze the data. Rates for survival, CD4 T cell count recovery and immune reconstitution cases in different patient groups by age, sex, behavior, clinical and treatment attributes was analyzed, Pearson’s Chi-square or Fisher’s exact test were used for the calculation of baseline characteristics with significant distribution. Hazard ratio (HR) was calculated using Wald test. Receiver operating characteristics (ROC) analysis was performed to estimate sensitivity of baseline CD4 T lymphocyte count for survival, CD4 T cell count recovery and immune reconstitution in different time of the ART. The Kaplan–Meier estimator was used for analyzing the survival function and estimating CD4 T cell recovery and immune reconstitution during the time of observation.

## Results

Data on survival, CD4 T cell count recovery, and immune reconstitution in patients who received first-line ART are shown in Table 3.

**Table 3:** Data on survival, CD4 T cell count recovery, and immune reconstitution in patients who received first-line antiretroviral therapy in Ulaanbaatar, Mongolia, during 2010–2017.

Survival, CD4 recovery and immune reconstitution	n/%
Patients enrolled	166/100.0
AIDS-related mortality for 3 year (36 months) of ART	9/5.4
AIDS-related mortality for first year (12 months) of ART	6/3.6
AIDS-related mortality for 6 months of ART	5/3.0
AIDS-related mortality for 3 months of ART	5/3.0
CD4 T cell count recovery during the ART (increased in >50 cell/μL),	
Recovery within first 3 months	104/62.7
Recovery within first year (12 months)	134/80.7
Recovery in the end of observation (36 months)	148/89.2
Immune reconstitution (reach the level >500 cell/μL)*	
With normal rate (>500 cell/μL) before initiation of the ART	21/12.7
Reconstitution, total (within 3 year)	95/65.5
Reconstitution within first 3 months	42/29.0
Reconstitution within first year (12 months)	69/47.6
No reconstitution during the ART	50/34.5

Abbreviations: AIDS, acquired immunodeficiency syndrome; ART, antiretroviral therapy

* calculated in 145 patients with baseline CD4 T cell count <500 cell/μL

### Association of the survival with baseline characteristics

Survival rate of the HIV-1 infected patients in the end point of observation or in the 36^th^ months of antiretroviral therapy in accordance to baseline characteristics defined before initiation of the therapy and shown in the Table 2 were analyzed (Table 4).

**Table 4:** Survival within 3 year of antiretroviral therapy according to baseline characteristics.

Baseline characteristics*		Mortality/Survival		Total	Statistics
		Dead	Live		
Clinical stage	Stage IV	9	29	38	Survival (stage IV) = 76.3%; p < 0.001
	Stage I—III	0	128	128	Survival (stage I—III) = 100.0%; p < 0.001
	Total	9	157	166	HR = 0.001 (0–39.4)^†^; p >0.05
CD4 T cell count (cell/μL)	< 200	9	32	41	Survival (<200) = 78.0%; p < 0.001
	≥200	0	125	125	Survival (≥200) = 100.0)^†^; p < 0.001
	Total	9	157	166	HR = 0.002 (0–17.0)^†^; p >0.05
Viral Load^‡^ (copy/mL)	≥500 000	3	13	16	Survival (≥500 000) = 81.2%; p < 0.05
	<500 000	3	92	95	Survival (<500 000) = 96.8%; p < 0.05
	Total	6	105	111	HR = 0.161 (0.033–0.80) ^†^; p <0.05

abbreviations: OR, odds ratio; HR, hazard ratio

*-only characteristics demonstrated significant survival rate are presented in the table (Fisher’s exact test, p < 0.05)

^†^-95% confidential interval for hazard ratio (Wald test)

^‡^-baseline viral load was available in 111 patients

Some baseline characteristics such as advanced disease stage, low count of peripheral blood CD4 T cells, and high replication activity before ART initiation may predict a higher risk for fatal outcomes and poor survival (Table 4).

**Fig 1:**
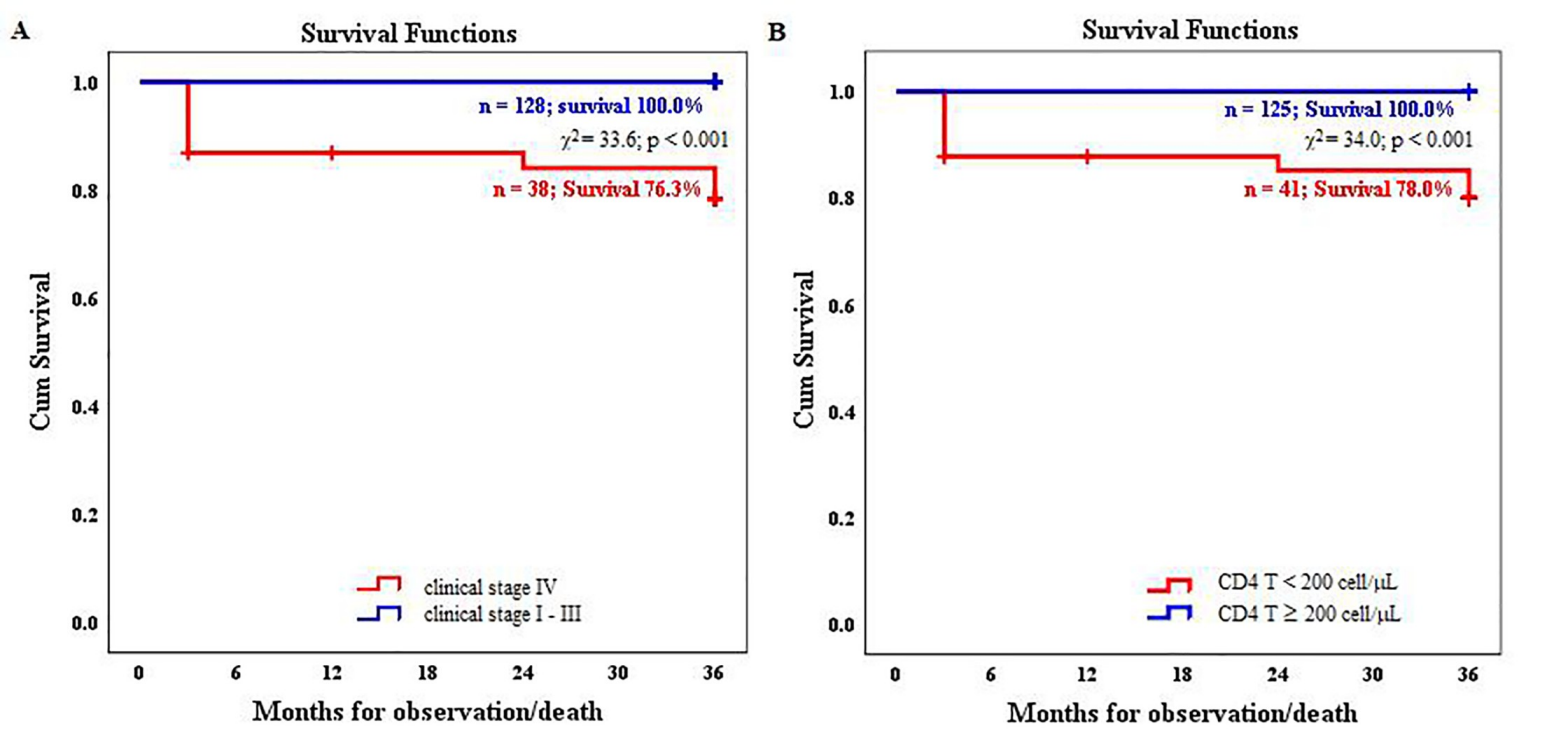
Survival of HIV-1 infected patients during the first-line antiretroviral therapy. (A) Survival function according to the clinical stage diagnosed before the initiation of antiretroviral therapy; and (B) survival function according to the baseline peripheral blood CD4 T cell count.

We analyzed the survival of HIV-1 infected patients who received first-line ART according to the baseline characteristics using the Kaplan–Meier estimator. The survival rates of patients with advanced stage (stage IV) HIV infection before ART initiation (Fig 1A) and patients with a low baseline CD4 T cell count (≤200 cells/μL; Fig 1B) significantly worsened during the therapy compared with those of patients with clinical stages I–III and patients with a higher baseline CD4 T cell count (>200 cells/μL), respectively.

### Association of the CD4 cell recovery with baseline characteristics

We analyzed the distribution of CD4 T cell count recovery state in HIV-1 infected patients at 12 months of ART according to the baseline characteristics. Some of the baseline characteristics were associated with a significantly lower recovery rate and PPV in women than in men (χ^2^ = 5.836; RR = 0.478; PPV = 15.4%). The recovery rate significantly increased in the third year (p<0.001) compared with that in the first year. Advanced pretreatment clinical stage, low baseline CD4 T cell count, and diagnosis of tuberculosis (TB) after ART initiation had a higher risk of non-responsiveness and lower response rate to ART (Table 5).

**Table 5:** CD4 T cell count recovery rates within 3 year of antiretroviral therapy according to baseline characteristics (an increase of >50 cells/μL).

Baseline characteristics*		Recovery		Total	Statistics
		Non-responder	Recovered		
Clinical stage	Stage IV	10	28	38	Recovery (stage IV) = 73.7%; p < 0.001
	Stage I—III	10	118	128	Recovery (stage I-III) = 92.2%; p < 0.001
	Total	20	146	166	HR = 1.4 (0.9–2.1)^†^; p > 0.05
CD4 T cell count (cell/μL)	< 200	10	31	41	Recovery (<200) = 75.6%; p < 0.01
	≥200	10	115	125	Recovery (≥200) = 87.9%; p < 0.01
	Total	20	146	166	HR = 1.37 (0.9–2.0)^†^; p >0.05
TB after initiation of ART^‡^	Yes	7	13	20	Recovery (TB) = 65%; p < 0.01
	No	13	124	137	Recovery (no TB) = 91.2%; p < 0.01
	Total	20	137	157	HR = 1.7 (1.0–3.0) ^†^; p <0.05

abbreviations: TB, tuberculosis; OR, odds ratio; HR, hazard ratio

*-only characteristics demonstrated significant difference in recovery rate are presented in the table (Fisher’s exact test, p < 0.05)

^†^-95% confidential interval for hazard ratio (Wald test)

^‡^-9 patients with TB detected before ART were excluded from analysis

Patients with an advanced pretreatment disease stage and a low count of CD4 T cells had a higher risk and lower probability of no response to ART compared with other patients (Table 5). Among the 18 patients diagnosed with TB, five were non-responders (OR = 3.410, RR = 1.244, p<0.05).

**Fig 2:**
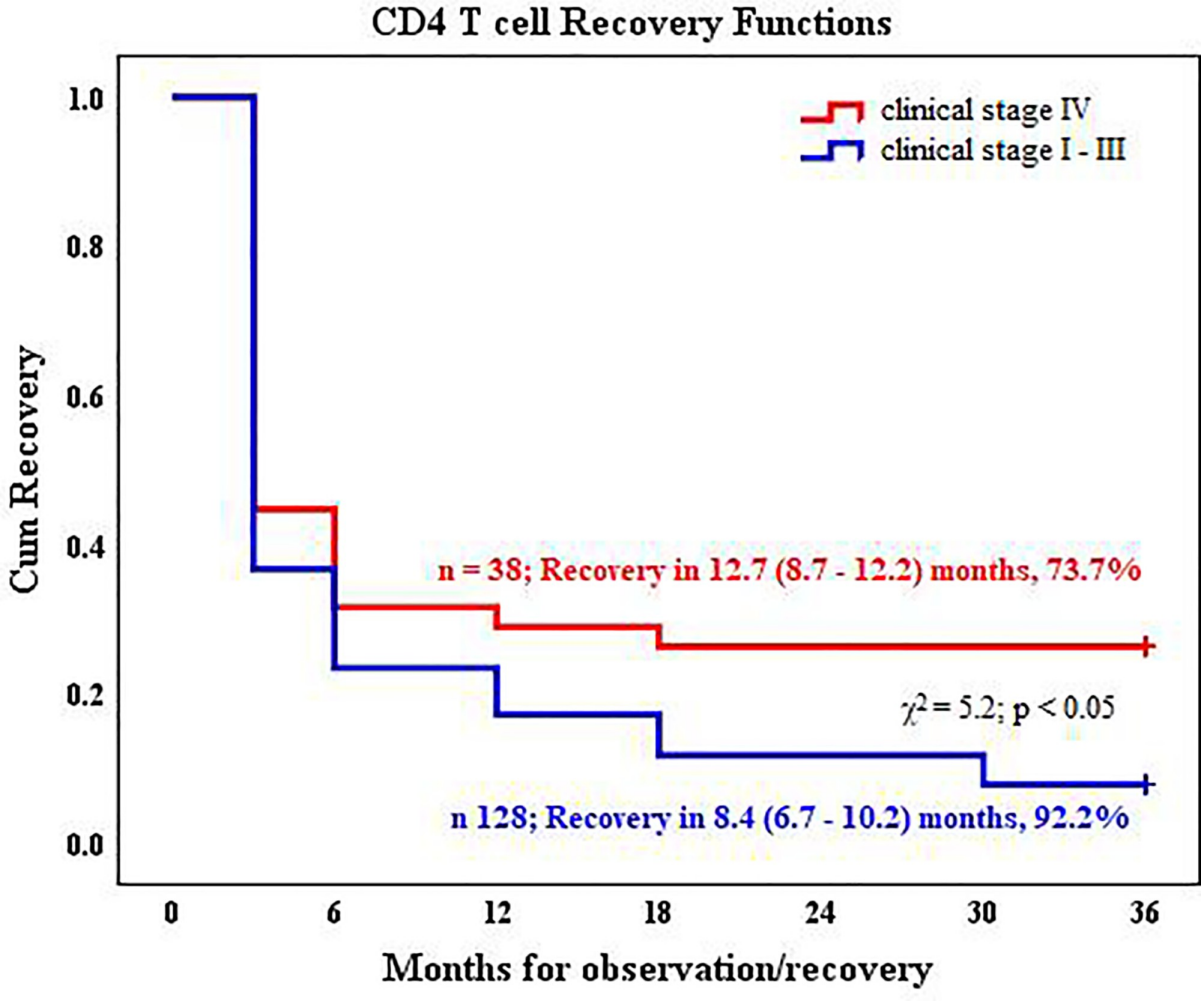
CD4 T cell count recovery during first-line antiretroviral therapy according to the clinical stage diagnosed before the initiation of the therapy in HIV-1 infected patient.

Patients with advanced clinical stage (stage IV) had a reduced CD4 T cell count recovery rate during ART compared with patients with clinical stages I–III (Fig 2).

### Association of the immune reconstitution with baseline characteristics

Significant differences were found in the distribution of immune reconstitution state (CD4 T cell count: >500 cells/μL) at different observational time-points according to the baseline CD4 T cell count, clinical stage, and viral load in 145 patients with a baseline CD4 count of <500 cells/μL. The relative risk and PPV of these baseline characteristics for the development of immune reconstitution were calculated in these patients (Table 6).

**Table 6:** Immune reconstitution rate and hazard ratio in patients with different baseline characteristics.

Baseline characteristics*	Within first 3 months of the ART		Within first year of the ART		Within end point of the observation	
	Immune^†^ reconstitution	HR^‡^ CI95 p	Immune reconstitution	HR^‡^ CI95 p	Immune^†^ reconstitution	HR^‡^ CI95 p
CD4 count						
<200 cell/μL	0%	2.8 (1.7–4.7)	14.6%	2.1 (1.5–3.0)	36.6%	1.9 (1.4–2.4)
200–350 cell/μL	30.0%		52.0%		77.5%	
351–500 cell/μL	50.0%		65.5%		83.3%	
p value	< 0.001	< 0.001	< 0.001	< 0.001	< 0.001	< 0.001
Clinical Stage						
Stage 1	30.2%	0.6 (0.4–0.8)	61.0%	0.7 (0.5–0.8)	74.4%	0.7 (0.6–0.8)
Stage II	37.5%		50.0%		87.5%	
Stage III	35.3%		58.8%		76.5%	
Stage IV	0.0%		13.5%		36.8%	
p value	< 0.05	< 0.005	> 0.05	< 0.001	< 0.05	< 0.005
Viral Load						
High (≥500 000 copies/mL)	12.5%	3.0 (0.7–12.7)	43.7%	1.3 (0.6–3.0)	62.5%	1.2 (0.6–2.4)
Low (<500 000 copies/mL)	37.9%		55.7%		69.6%	
p value	< 0.05	> 0.05	> 0.05	> 0.05	> 0.05	> 0.05

abbreviations: ART, antiretroviral therapy; HR, hazard ratio; CI95, 95% confidential interval

*-only characteristics with significant difference in immune reconstitution rate are presented in the table (Fisher’s exact test, p < 0.05)

^†^-p values for immune reconstitution rate were calculated using Pearson’s chi-square

-p values for hazard ratio were calculated using Wald test

No or a low capacity for the development of complete immune reconstitution was observed in patients with advanced stages of the disease during ART, especially in the early stages of ART.

By contrast, patients with early stages of the disease (stage I) and a higher count of CD4 T cells (350–500 cells/μL) had a greater possibility of being cured from immune suppression and the possibility gradually increased during ART.

**Fig 3:**
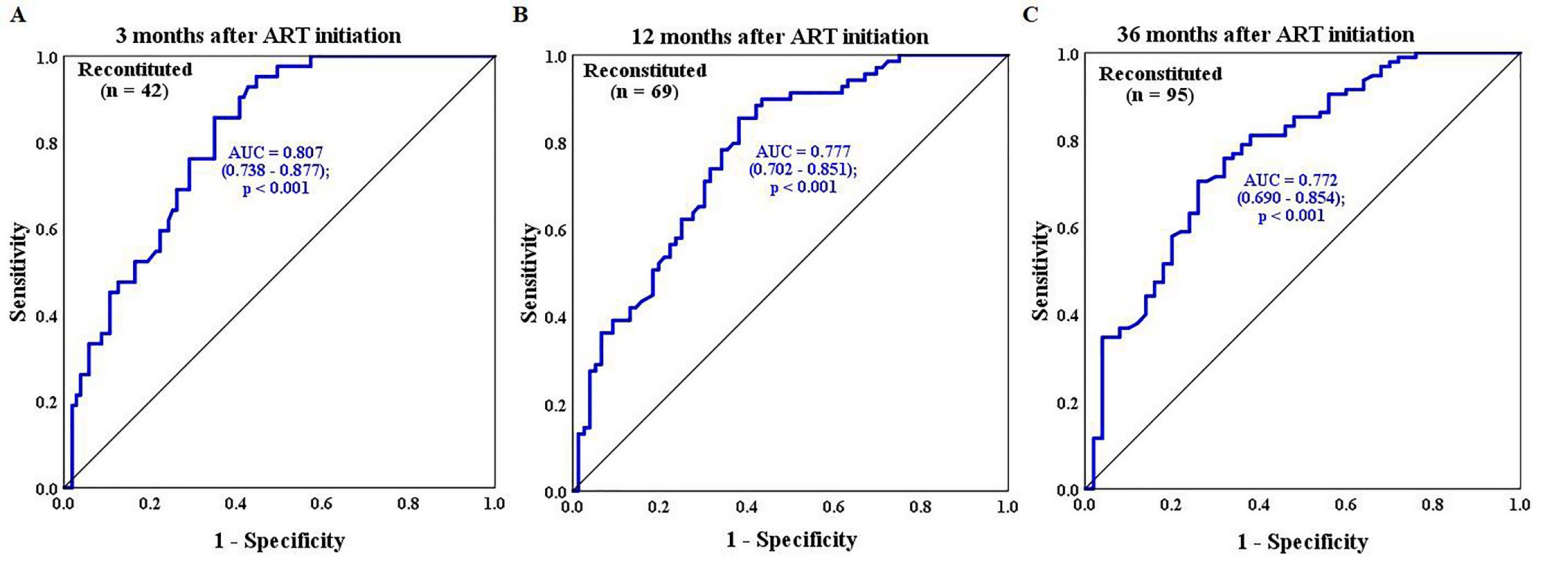
Receiver operating characteristic analysis. **Sensitivity and specificity of the baseline CD4 T cell count for the development of immune reconstitution in human immunodeficiency virus-1 infected patients.** (A)—Immune reconstitution in the first 3 months; (B) Immune reconstitution in the first year; and (C) Immune reconstitution at the end-point (36 months) of antiretroviral therapy (C). AUC–Area under the curve.

Receiver operating characteristic (ROC) analysis revealed that the baseline CD4 T cell count was a sensitive predictive value for immune reconstitution (Fig 3), but not for survival or CD4 T cell count recovery.

**Fig 4:**
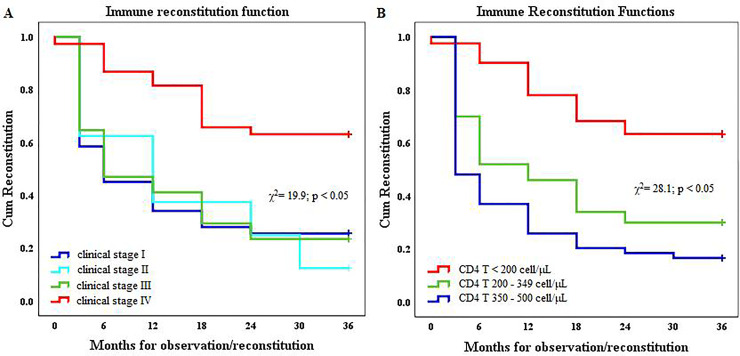
Immune reconstitution during antiretroviral therapy in human immunodeficiency virus-1 infected patients. (A) Immune reconstitution according to the disease stage; and (B) Immune reconstitution function according to the baseline CD4 T cell count.

Immune reconstitution function during the ART in patients with baseline immunosupression was differentiated according to disease stage (Fig 4A) and baseline CD4 T cell count (Fig 4B).

## Discussion

An advanced pretreatment clinical stage of HIV infection (as classified by the WHO classification), a low CD4 T cell count in the peripheral blood, and a high viral load before the initiation of first-line ART were found to be accurate predictive factors for survival, CD4 T cell count recovery, and immune reconstitution in Mongolian patients with HIV/AIDS.

### Survival/mortality and baseline characteristics

In this study, the 3- and 1-year mortality rates were 5.4% (survival rate: 94.6%) and 3.6%, respectively. A total of 83% of deaths that occurred in the first year occurred within the first 3 months. The 1-year mortality rate in our study was lower than that (8.3%; range: 7.6%–9.1%) in a South American multinational study (1996–2007), which reported that 80% of the AIDS-related deaths that occurred in the first year occurred within the first 6 months [14]. The first year survival rate was lower in our cohort (Mongolian patients) than in the Chinese cohort (96.4 vs. 97.1) [15]; however, the survival rate at the end of the third year was higher in our cohort than in the Chinese cohort (94.6 vs. 90.6). Additionally, the third year survival rate in our cohort was similar to the fourth year survival rate (94.5%) in seven Asia-Pacific regions in 2010–2013 [16]. In our study, 55.5% of the deaths occurred within the first 6 months of ART. A number of studies have reported higher [14], lower [15], and similar [17] proportions of deaths compared with our study. Several studies have reported that older age [17, 18], male gender [15, 17], low baseline count of CD4 T cell [14–17, 19–23], advanced clinical stage [17], high viral load [21, 22, 24], and TB co-infection [17] are associated with a higher risk of death. However, our study revealed that sex and sexual behavior were not associated with ART outcome.

### CD4 T lymphocyte count recovery

The CD4 T cell count recovery (increase of >50 cells/μL) rate at 3, 12, and 36 months of ART were 62.7%, 80.7%, and 89.2%, respectively. The 1- and 3-year non-response rates were lower in our cohort (19.3 and 10.8%, respectively) than in an Ethiopian cohort (22.1% and 13.8%, respectively) [34]. Regarding CD4 T cell count recovery, non-response to ART at the end-point of the observation was found to be associated with a low baseline CD4 T cell count and advanced pretreatment clinical stage. Non-responders were at an increased risk of developing pulmonary TB. Our findings were similar to those of other studies that reported an association between non-response to ART or CD4 T cell count recovery function and the baseline CD4 T cell count [34–39] and advanced clinical stage [34, 38]; however, non-response was not associated with the baseline viral load [35, 39], ARV combination, and TB co-infection [34]. A number of studies have reported poor response rates in men[16, 36, 39]. However, the reason for the poor response to ART during the first 12 months in women in our study is unclear because no differences were found in the baseline characteristics of these patients.

### Immune reconstitution

The CD4 T cell count increased to >500 cells/μL in 65.5% (95/145) of patients with a baseline cell count of <500 cell/μL after 36 months of ART initiation in our study. The immune reconstitution rate was higher than that (47.6% at 60 months) in an early Swiss HIV cohort [40] and lower than the 5-year rate (72%) in an Australian cohort [41]. The reason for immunological non response is incompletely understood, and approximately 20% of all HIV-infected patients do not achieve optimal immune reconstitution despite the suppression of viral replication [42].

Some studies have reported delayed and low rate of immune reconstitution during ART, especially in early periods of the therapy in patients with advanced stages of the disease [30, 43]. The baseline CD4 T cell count was found to be a sensitive indicator for immune reconstitution in this study, which is consistent with the findings of another study [44]. The association of the baseline CD4+T cell count with immune reconstitution after ART initiation has been reported by several studie [37, 40, 41, 43–45].

We found that a high viral load (>100,000 copies/mL) before ART initiation was associated with incomplete immune reconstitution. Although several studies have reported the association between the pretreatment viral load and immune reconstitution in patients receiving ART [46], some studies have reported no association between them [47].

## Study limitation

We found a strong association between ART outcome and the baseline CD4 count and pretreatment clinical stage of HIV/AIDS. The small number of patients we observed and the short observation period may not have allowed us to find as many predictive values as described in long-term studies performed in a large population. Furthermore, data on reduction of viral replication during therapy were insufficient for accurate analysis.

## Conclusions

An advanced pretreatment clinical stage of HIV infection (as classified by the WHO classification), a low CD4 T cell count in the peripheral blood, and a high viral load before the initiation of first-line ART were found to be accurate predictive factors for survival, CD4 T cell count recovery, and immune reconstitution in Mongolian patients with HIV/AIDS.

## Supporting information

S1 Questionnaire (DOCX)

S2 Questionnaire (DOCX)
