# Correction: Survival, CD4 T lymphocyte count recovery and immune reconstitution pattern during the first-line combination antiretroviral therapy in patients with HIV-1 infection in Mongolia

**DOI:** 10.1371/journal.pone.0269054

**Published:** 2022-05-23

**Authors:** Solongo Bayarsaikhan, Davaalkham Jagdagsuren, Batbaatar Gunchin, Tsogtsaikhan Sandag

There are errors in the author affiliations. The order of affiliations 1 and 2 are incorrect. The correct affiliations are as follows:

Solongo Bayarsaikhan^1,2^, Davaalkham Jagdagsuren^2^, Batbaatar Gunchin^1^, Tsogtsaikhan Sandag^1^

**1** Department of Immunology, School of Biomedicine, [1] Mongolian National University of Medical Sciences, Ulaanbaatar, Mongolia, **2** AIDS/ STI Research and Surveillance Division, National Center for Communicable Diseases, MoH, Ulaanbaatar, Mongolia.
