# Supplementary material for: Survival, CD4 T lymphocyte count recovery and immune reconstitution pattern during the first-line combination antiretroviral therapy in patients with HIV-1 infection in Mongolia
Source: PLoS One. 2021 Mar 8;16(3):e0247929. doi: 10.1371/journal.pone.0247929 (PMC7939265; doi:10.1371/journal.pone.0247929)
Supplement: S1 Questionnaire — (DOCX) [file pone.0247929.s001.docx]

**Registration form**

**Female sex workers**

**Interview date:**

**Day** **Month** **Year**

|  |  |  |  |  |  |  |  |
| --- | --- | --- | --- | --- | --- | --- | --- |

**Interviewee code Interviewer code**

| **Aimag and city code** (2 digit)  01= Ulaanbaatar  02= Darkhan  03= Dornod  04= Khuvsgul | **UB city district code**  01 = Bayangol  02 = Bayanzurkh  03 = Songino-Khairkhan  04 = Sukhbaatar  05 = Khan-Uul  06 = Chingeltei | **Location code**  01=Bar  02=Hotel  03=Sauna  04=Massage  05=Street  06=NGO activities  07=Home  08=Karaoke |
| --- | --- | --- |

**Questions to check eligibility of the participant**

**Question 1:** Have you been interviewed 1Yes 2No

for this study in the past 3 months?

**Question 2:** How old are you? 1 Yes2 No

(Include only women aged from 15 to 49)

**Question 3:** Have you exchangedgift/money

for sex in the last12 months? 1 Yes 2No

**Accepted for participation in the survey**

1= Accepted (Proceed) 2= Not accepted (Do not proceed)

why are you refused to participate the survey.

01=Busy, with customer

02=boss do not accept

03=other, please write

**Consent of the participant**  If participant provided consent, tick √ the appropriate box.

If refused, do not proceed.

**Interviewer’s note:**

1. Completed Registration form: 1. Yes 2. No

2. Interviewee’s code on consent form, Questionnaire, Registration form, blood collection tube and Laboratory registration are same: 1. Yes 2. No

3. All questions have been answered: 1. Yes 2. No

4. Answers are related to each other: 1. Yes 2. No

5. Interviewee’s Questionnaire, Registration form are clipped together: 1. Yes 2. No

**Interviewer’s signature: ________________________________ Date: year_____month_____day_____**

**Sexual Behavior Survey Questionnaire**

**Female Sex Workers**

|  |  |  |  |  |  |  |  |
| --- | --- | --- | --- | --- | --- | --- | --- |

Participant code:

**Section 1: General information**

| **№** | | **Question** | **Code** | **Answer** |
| --- | --- | --- | --- | --- |
|  | | Date of your birth? | Record year of birth (4 digits) | \|__\|__\|__\|__\| |
|  | Residency | | 01 = Ulaanbaatar  02 = Darkhan Uul  03 = Orkhon  04 = Other (_______________________note) | \|__\|__\| |
|  | What is your education Level? | | 00= None  01= Primary/1-4grade/  02 =Incomplete secondary/5-8 grade/  03= Complete secondary/9-11 grade/  04= Special Vocational School, College  05= University (at least bachelor degree)  88 = Don’t want to answer | \|__\|__\| |
|  | Aside from selling sex for money or goods, do you have other employment? | | 01=Yes  02=No (if answer is no, please move to 1.6) |  |
|  | What ways, other than selling sex, do you earn money | | 01=Laborer/Maid/Cleaner  02=Beautician, massage, salon  03=Selling things/informal work  04=Full time office work  05=Part time office work  06=Entreprenuer/business person  07=Hotel/restaurant  08=NGO worker  09=Other (write): | \|__\|__\| |
|  | Marital Status? | | 01= Married  02= Not married  03 = Widowed  04 = Divorced | \|__\|__\| |
|  | Do you currently live with your sexual partner? | | 01=Yes  02=No | \|__\|__\| |

**Section2: Sexual behavior**

| **№** | **Question** | **Code** | | | | | | | | | **Answer** | |
| --- | --- | --- | --- | --- | --- | --- | --- | --- | --- | --- | --- | --- |
|  | At what age did you have sexual intercourse (vaginal or anal)? | Age  88 = Don’t want to answer  99 = Don’t remember | | | | | | | | | \|__\|__\| | |
|  | At what age did you start selling sex? | Age  88 = Don’t want to answer  99 = Don’t remember | | | | | | | | | \|__\|__\| | |
|  | What are the reasons you sell sex? (Multiple responses possible) | 01 = Can make more money  02=I have no other skills  03=To help family (food, medical expenses, housing, etc.)  04 = To pay off debts  05 = I am being forced  06 = In order to earn a livelihood for my children  07 = To pay off tuition  08 = To have a sexually active relationship  09 = Other (_______________________note) | | | | | | | | | \|__\|__\| | |
|  | How many days have you worked exchanging sex for money or goods within last week | Number of days  00= None  88= Don’t want to answer  99=Don’t know | | | | | | | | | \|__\|__\| | |
|  | How many months have you worked as paid sex worker in the last 12 months? | Months  88 = Don’t want to answer  99 = Don’t remember | | | | | | | | | \|__\|__\| | |
| **If you don’t use condoms at all, skip 2.6**  **If you regularly use condoms, skip 2.7** | | | | | | | | | | | | |
|  | What is your income from sex, when a condom is used? *(convert to cash amount if items are received instead)* | Per hour   \|  \|  \|  \|  \|  \|  \| \| --- \| --- \| --- \| --- \| --- \| --- \|   Per day   \|  \|  \|  \|  \|  \|  \| \| --- \| --- \| --- \| --- \| --- \| --- \| | | | | | | | | | \|__\|__\| | |
|  | What is your income from sex, when a condom is not used? *(convert to cash amount if items are received instead)* | Per hour   \|  \|  \|  \|  \|  \|  \| \| --- \| --- \| --- \| --- \| --- \| --- \|   Per day   \|  \|  \|  \|  \|  \|  \| \| --- \| --- \| --- \| --- \| --- \| --- \| | | | | | | | | | \|__\|__\| | |
|  | On the last day you worked, how many clients did you have sexual intercourse with? | Number of partners  88 = Refuse to answer  99 = Don’t know | | | | | | | | | \|__\|__\| | |
|  | Have you ever had anal sex? | 01=Yes  02=No  88=Don’t want to answer | | | | | | | | | \|__\|__\| | |
|  | Have you ever sold sex in a country other than Mongolia? | 01=Yes  02=No  88=Don’t want to answer | | | | | | | | | \|__\|__\| | |
|  | How do you find clients?  *(Multiple answers possible)* | 01= Over internet and social network (i.e. Facebook)  02= Through calls  03 = Through friends’ network  04= Through an intermediary  05= Public events  06= Through work stations (Sauna, massage parlors, receptions, bars, hotels, karaoke)  07= Public places (streets, parking lots, parks)  08= Other (_______________________note)  88= Don’t want to answer | | | | | | | | | \|__\|__\| | |
|  | Where do you mainly find clients?  *(Multiple answers possible)* | 01=Bar/karaoke  02=Hotel/motel  03=My own home  04=Street and parks  05=Sauna, massage parlors  06=Client’s home  07=Friends’ home  08=Other (_______________________note)  88= Don’t want to answer | | | | | | | | | \|__\|__\| | |
|  | About sexual intercourse | | | | | | | | | | | |
|  |  | Types of partner (Last 12 months) | | | | Number of partners (last month) | | Condom used at last sex with partner type (in last 12 months) | | | | |
|  | Type of sexual partner | Yes | | No | | Number | | Yes | | | No | |
|  | Steady partner (person you were/ are in a relationship) | 1 | | 2 | | \|__\|__\| | | 1 | | | 2 | |
|  | Non-permanent, casual sex partner who doesn’t pay for sex | 1 | | 2 | | \|__\|__\| | | 1 | | | 2 | |
|  | A man who paid you *(someone who paid you money, goods, or resources other than money for sexual intercourse)* | 1 | | 2 | | \|__\|__\| | | 1 | | | 2 | |
|  | In the last 12 months, how often did you use a condom during sex? | | | | | | | | | | | |
|  |  | Steady partner (person you were/ are in a relationship) | | | Non-permanent, casual sex partner who doesn’t pay for sex | | | | A man who paid you | | | |
|  | Always (100%) | 1 | | | 1 | | | | 1 | | | |
|  | Most of the times (75%) | 2 | | | 2 | | | | 2 | | | |
|  | About half of the time (50%) | 3 | | | 3 | | | | 3 | | | |
|  | Sometimes (25%) | 4 | | | 4 | | | | 4 | | | |
|  | Rarely (10%) | 5 | | | 5 | | | | 5 | | | |
|  | Never | 6 | | | 6 | | | | 6 | | | |
|  | What were the reasons of you having sex without condom?*There may be multiple choices selected* (for each sexual partner) | | | | | | | | | | | |
|  |  | Non-permanent, casual sex partner who doesn’t pay for sex | | | Steady partner (person you were/ are in a relationship) | | | | A man who paid you | | | |
|  |  | Yes | No | | Yes | | No | | | Yes | | No |
|  | 01 = I do not think sex feels good with a condom | 1 | 2 | | 1 | | 2 | | | 1 | | 2 |
|  | 02 = Forget to use | 1 | 2 | | 1 | | 2 | | | 1 | | 2 |
|  | 03 = Didn’t find any | 1 | 2 | | 1 | | 2 | | | 1 | | 2 |
|  | 04 = There was no time to buy one | 1 | 2 | | 1 | | 2 | | | 1 | | 2 |
|  | 05 = Expensive | 1 | 2 | | 1 | | 2 | | | 1 | | 2 |
|  | 06 = It’s not comfortable to buy, it’s embarrassing | 1 | 2 | | 1 | | 2 | | | 1 | | 2 |
|  | 07 = I don’t trust condoms | 1 | 2 | | 1 | | 2 | | | 1 | | 2 |
|  | 08 = I use other methods to prevent HIV/STIs | 1 | 2 | | 1 | | 2 | | | 1 | | 2 |
|  | 09 = My partner doesn’t like | 1 | 2 | | 1 | | 2 | | | 1 | | 2 |
|  | 10 = I trust my partner | 1 | 2 | | 1 | | 2 | | | 1 | | 2 |
|  | 11 = Can’t get enough condoms | 1 | 2 | | 1 | | 2 | | | 1 | | 2 |
|  | 12 = I was drunk | 1 | 2 | | 1 | | 2 | | | 1 | | 2 |
|  | 13 = Customer was drunk | 1 | 2 | | 1 | | 2 | | | 1 | | 2 |
|  | 14 = Violent matter | 1 | 2 | | 1 | | 2 | | | 1 | | 2 |
|  | 15 = Get paid more without condom | 1 | 2 | | 1 | | 2 | | | 1 | | 2 |
|  | 16 = Use other contraceptives | 1 | 2 | | 1 | | 2 | | | 1 | | 2 |
|  | 17=Want to get pregnant | 1 | 2 | | 1 | | 2 | | | 1 | | 2 |
|  | 18=My partner and I are seropositive | 1 | 2 | | 1 | | 2 | | | 1 | | 2 |
|  | 19 = They’ll think I have STI | 1 | 2 | | 1 | | 2 | | | 1 | | 2 |
|  | 20 = Other ……………..(note) |  |  | |  | |  | | |  | |  |
|  | Can you get condoms every time you need a condom? | 01 = Yes  02 = No(if no, move to 2.18)  03 = I’ve never had any need  04 = I don’t use condoms | | | | | | | | | \|__\|__\| | |
|  | Where do you get condoms when you need one? (DO NOT READ RESPONSE. LET RESPONDENT RESPOND AND THEN ASK, **“ANYTHING ELSE?”** MULTIPLE RESPONSES POSSIBLE. NEED TO CIRCLE **YES** OR **NO** FOR EACH RESPONSE) | | | | | | | | | | | |
|  |  | Yes | | | | | No | | | | | |
|  | 1. Pharmacy | 1 | | | | | 2 | | | | | |
|  | 2. From the sex partner | 1 | | | | | 2 | | | | | |
|  | 3. NCCD | 1 | | | | | 2 | | | | | |
|  | 4. Zaluus Eruul Mend NGO | 1 | | | | | 2 | | | | | |
|  | 5. From the an outreach NGO worker | 1 | | | | | 2 | | | | | |
|  | 6. District Health Center | 1 | | | | | 2 | | | | | |
|  | 7. Other............................ (note) | 1 | | | | | 2 | | | | | |
|  | Why aren’t you able to buy a condom when you need one? (DO NOT READ RESPONSE. LET RESPONDENT RESPOND AND THEN ASK, **“ANYTHING ELSE?”** MULTIPLE RESPONSES POSSIBLE. NEED TO CIRCLE **YES** OR **NO** FOR EACH RESPONSE) | | | | | | | | | | | |
|  |  | Yes | | | | | No | | | | | |
|  | 1. Expensive | 1 | | | | | 2 | | | | | |
|  | 2. Pharmacy is too far | 1 | | | | | 2 | | | | | |
|  | 3. Pharmacies are closed when I need a condom | 1 | | | | | 2 | | | | | |
|  | 4. Too embarrassed to buy a condom | 1 | | | | | 2 | | | | | |
|  | 5. I don’t know where to buy one | 1 | | | | | 2 | | | | | |
|  | 6. I dislike carrying a condom | 1 | | | | | 2 | | | | | |
|  | 7. Other....................... (note) | 1 | | | | | 2 | | | | | |
|  | Do you know of any organizations that offer free condoms and other services? | 01= Yes  02= No | | | | | | | | | \|__\|__\| | |

**Section 3: Knowledge and attitudes of HIV/AIDS and voluntary counseling and testing of HIV/AIDS**

| **№** | **Асуулт** | **Код** | **Хариулт** |
| --- | --- | --- | --- |
|  | Have you ever heard about HIV/AIDS? | 01= Yes  02= No (if answer is no, please move to 3.8) | \|__\|__\| |
|  | Do you think that having one, faithful sex partner can reduce transmission risk of HIV infection? | 01= Yes  02= No  99 = Don’t know | \|__\|__\| |
|  | Do you think that using condom properly in all sexual intercourses can prevent HIV infection? | 01= Yes  02= No  99 = Don’t know | \|__\|__\| |
|  | Do you think healthy looking person can be infected with HIV? | 01= Yes  02= No  99 = Don’t know | \|__\|__\| |
|  | Which type of sex has the highest risk of HIV?  *(Choose one answer only)* | 01 = Vaginal  02 = Anal  03 = Oral  04 = Other (_______________________note)  99 = Don’t know | \|__\|__\| |
|  | What do you consider your risk for contracting HIV to be? | 01=no risk  02=low risk  03=medium risk  04=high risk  05=very high risk  06=I already have HIV | \|__\|__\| |
|  | Do you know where to have an HIV test? | 01= Yes  02= No  99 = Don’t know | \|__\|__\| |
|  | Have you ever tested for HIV? | 01= Yes  02= No (if answer is no, please move to 3.14) | \|__\|__\| |
|  | In last time Have you tested for HIV infection voluntarily or was it required? (e.g. having surgery, applying for a job, going abroad) | 01= Voluntarily  02= Required  03 = Provider initiated | \|__\|__\| |
|  | When was your last HIV tested | 01= 6 months  02=6-12 months  03=More than 12 months  04=refuse to answer | \|__\|__\| |
|  | Did you receive your test results last time you were tested? | 01= Yes  02= No (if answer is no, please move to 3.14) | \|__\|__\| |
|  | Can you please tell me the result of your last test? | 01= Positive  02= Negative  03= Indeterminate  88 = Don’t want to answer | \|__\|__\| |
|  | If yes, have you received antiretroviral treatment? | 01= Yes  02= No | \|__\|__\| |
|  | Do you agree that having regular screening (every 3-6 months) for HIV/STI will help you and your clients prevent the risks of infection? | 01=Yes  02=No  99=Don’t know | \|__\|__\| |
|  | How many times have you enrolled/participated in HIV prevention activities organized by the “Perfect Lady” NGO in last 12 months? *(if no, write down 00)* | Number  99=Do not know | \|__\|__\| |
|  | Which of the following services have you received from an outreach service, drop-in centre or sexual health clinic in the past 3 months?(DO NOT READ RESPONSE. LET RESPONDENT RESPOND AND THEN ASK, **“ANYTHING ELSE?”** MULTIPLE RESPONSES POSSIBLE. NEED TO CIRCLE **YES** OR **NO** FOR EACH RESPONSE) | | |
|  |  | Yes | No |
|  | 1.Public events (events, trainings) | 1 | 2 |
|  | 2.Tests for infections that are spread through sexual contact (sexually transmitted infections) | 1 | 2 |
|  | 3.Counselling on condom use and safe sexual practices | 1 | 2 |
|  | 4.Free condoms, lubricants | 1 | 2 |
|  | 5.Other ………………………*(note)* | 1 | 2 |

**Section 4: Knowledge and attitude of Sexually transmitted infections (STIs)**

| **№** | **Question** | **Code** | | **Answer** |
| --- | --- | --- | --- | --- |
|  | Have you had a genital discharge in the last 12 months? | 01 = Yes  02 = No | | \|__\|__\| |
|  | If yes, did I seek treatment? | 01 = Yes  02 = No (if answer is no, please move to 4.4) | | \|__\|__\| |
|  | If yes, where did you receive treatment? | 01= NCCD, AIDS Unit  02= District, aimag hospital  03= Private hospital  04 = NGO  05 = Other……………….…*(note)* | | \|__\|__\| |
|  | Have you been tested for STI in the last 12 months? | 01=Yes  02=No (if answer is no, please move to 5.1) | | \|__\|__\| |
|  | If yes, what were the reasons for getting tested? | 01=As a preventative measure, on your own 02=As a preventative measure, as a part of a project 03=Experienced the symptoms  04=Because of an unprotected sex  05=Because sexual partner requested it  06=Other……………….…*(note)* | | \|__\|__\| |
|  | What kind of difficulties do you experience when getting tested for STIs other than HIV? | | | |
|  |  | Yes | No | |
|  | Waited for too long | 1 | 2 | |
|  | Work hours of a clinic are unsuitable | 1 | 2 | |
|  | Lack of privacy | 1 | 2 | |
|  | Negative attitude of the hospital workers (offensive, discriminating) | 1 | 2 | |
|  | Hospital environment is uncomfortable (not enough chairs, unhygienic, lack of privacy) | 1 | 2 | |
|  | The hospital was too far | 1 | 2 | |
|  | The service fee is too high | 1 | 2 | |
|  | Other……………….…*(note)* | 1 | 2 | |
|  | Were you diagnosed with any of the following in the last 12 months? | | | |
|  |  | Yes | No (if no, move to 5.1) | |
|  | Syphilis | 1 | 2 | |
|  | Gonorrhoea | 1 | 2 | |
|  | Chlamydia | 1 | 2 | |
|  | If yes, did you receive a treatment? | 01 = Yes  02 = No (if answer is no, please move to 4.12) | | \|__\|__\| |
|  | If yes, who treated you? | 01= Doctor  02=Pharmacist  03=Self-treated 04= Other……………….…*(note)* | | \|__\|__\| |
|  | If not, why did not you seek treatment? *(there may be multiple answers)* | 01 = Symptoms disappeared  02 = Ashamed from other people  03 = Afraid from the doctor  04 = There was no money  05 = Could not find a suitable clinic  06 = Did not know where to approach  07 = No time for approach public clinic  08=Other*………………........……..........(write)* | | \|  \|  \| \| --- \| --- \| \|  \|  \| \|  \|  \| \|  \|  \| \|  \|  \| \|  \|  \| \|  \|  \| |

**Section5: Drug and alcohol use**

| **№** | **Question** | **Code** | **Answer** |
| --- | --- | --- | --- |
|  | Have you consumed alcoholic beverages in the past 12 months? | Yes  No (skip to 5.4) |  |
|  | How often in the past 12 months did you consume alcoholic beverages? | 01 = Once a week  02 = More than once a week  03 = Once a month  04 = More than once a month  05 = Once or twice a year  06 = Do not drink at all | \|__\|__\| |
|  | In the past year, have you used alcoholic beverages before having sex? | 01 = Yes  02 = No(skip to 5.5) | \|__\|__\| |
|  | In the past year, have often did you use alcoholic beverages before having sex? | 01=Always (100%)  02=Usually (75%)  03=Sometimes (50%)  04=Rarely (10%)  88= do not answer | \|__\|__\| |
|  | Have you ever used illicit non-injectable drugs to get high? | 01= Yes  02= No (if answer is no, please move to 6.1) | \|__\|__\| |
|  | If yes, what kind of non-injecting drugs have you used? (multiple choices) | 1 = Marijuana/Hashish  2 = Cannabis  3 = Ecstasy  4 = Vaporizing substances (glue, acetone, benzine)  5 = Amphetamines  6 = Heroine  07 = Methadone  08 = Cocaine  09=Ice  10=Drugs  11=Mimi  12=Popers  13=  14 = Tranquilizers or sedatives (diazepam, valium, tranqwin, lorazepam, seduxen, tazepam, oxazepam) | \|__\|__\| |
|  | Have you ever injected drugs to get high? | 01= Yes  02= No (if answer is no, please move to 5.10) | \|__\|__\| |
|  | If yes, what kind of injecting drugs have you used? | 01 = Heroine  02 = Cocaine  03 = Morphine  04 = Amphetamines  05 = Prescription drugs (OxyContin, Vicodin, Valium, Alprazolam, Adderall, Ritalin etc.)  06 = Other*………………........……..........(write)* | \|__\|__\| |
|  | If yes, have you ever shared a syringe or needle to inject drugs? | 01 = Yes  02 = No  99= Don’t know | \|__\|__\| |

**Section 6:Discrimination and violence**

| **№** | **Question** | | | | **Code** | | | | | **Answer** | |
| --- | --- | --- | --- | --- | --- | --- | --- | --- | --- | --- | --- |
|  | Who knows about your sexual work? *“Multiple answers possible".* | | | | 01 = Family members  02 = Close friends  03 = Relatives  04 = Colleagues  05 = Only my sexual partner 06 = Other (_______________________note) | | | | | \|__\|__\|  \|__\|__\|  \|__\|__\|  \|__\|__\|  \|__\|__\| | |
|  | Have you ever been physically attacked because you sell sex for money or goods? | | | | 01=Yes, once02=Yes, multiple times03=No04=Refuse answer | | | | | \|__\|__\| | |
|  | Have you ever been exposed to violence or abuse from the police because sell sex for money or goods? | | | | 01=Yes, once02=Yes, multiple times03=No04=Refuse answer | | | | | \|__\|__\| | |
|  | Have you ever been denied health or medical services because you sell sex for money or goods? | | | | 01=Yes, once02=Yes, multiple times03=No 04 =Refuse answer | | | | | \|__\|__\| | |
|  | Have you ever been worried or afraid of getting the following services in the last 12 months? | | | | | | | | | | |
|  | Service type | Afraid of stigma | | Afraid of someone finding out about my sexual orientation | | | Was attacked before/Afraid of violence | | Was arrested by the police before/Afraid of being arrested | | |
|  |  | Yes | No | Yes | | No | Yes | No | Yes | | No |
|  | Health services | 1 | 2 | 1 | | 2 | 1 | 2 | 1 | | 2 |
|  | HIV screening test | 1 | 2 | 1 | | 2 | 1 | 2 | 1 | | 2 |
|  | Have you ever been in arrested? | | | | 01=Yes, once02=Yes, multiple times03=No 04 =Refuse answer | | | | | \|__\|__\| | |
|  | Has a man ever forced you to have sex with him? | | | | 01= Yes  02= No  03 =Refuse answer | | | | | \|__\|__\| | |
|  | In the past 12 months, have you ever wanted to use a condom during anal/vaginal sex and the partner refuse? | | | | 01= Yes  02= No (if answer is no, please move to 7.1)  03 =Refuse answer | | | | | \|__\|__\| | |
|  | Did you still have sexual intercourse with a partner who refused to use a condom when you wanted to use one in the past 12 months? | | | | 01= Yes  02= No  03 =Refuse answer | | | | | \|__\|__\| | |

**Section 7**: **Size estimaton**

**Size Estimation.** Questions for RDS/TLS for Multiplier Population Size Estimation

| **NO.** | **QUESTIONS AND FILTERS** | **CODING CATEGORIES** | | **SKIP** |
| --- | --- | --- | --- | --- |
| **Unique object multiplier** | |  | |  |
| SE1 | Did you receive a medicine storage in the recent past? (Prompt if not willing to say/doesn’t remember) | Yes 1  No 2 | | 🡺SE5 |
| SE2 | Which is the medicine storage you received? (show picutre of different key rings) | Yes 1  No 2 | | 🡺SE5 |
| SE3 | How many medicine storage did you receive? | **\|____\|____\|** | |  |
| SE4 | When did you receive this medicine storage?  Must be around (enter distribution dates) 2019 | _ _/ _ _ _ _ (mm/yyyy) | |  |
| SE5 | **Interviewer:** Did participant receive a medicine storage? | Yes 1  No 2 | |  |
| **Service multiplier** | |  | |  |
| SE6 | Between April 4 and June 30, did you get an HIV test at NGO named “Perfect Lady”? | Yes 1  No 2 | |  |
| SE7 | Between April 4and June 30, did you get free condoms at NGO named “Perfect Lady”? | Yes 1  No 2 | |  |
|  | Between April 4and June 30, did you enrolled behavior change communication program at NGO named “Perfect Lady”? | Yes 1  No 2 | |  |
| **App service multiplier** | |  | |  |
| SE8 | From X 2019 to X 2019 (one month before survey) did you use any of the following apps for the purposes of meeting other men for sex? How many different times during the month did you log on to the app? | APP  GRINDR  FACEBOOK  More | TIME LOGGING IN  ___  ___ |  |
| **Wisdom of the crowds for FSW** | |  |  |  |
| SE9 | What is your best guess of the maximum number of women who have willingly sold vaginal or anal sex for money or favors in the past 12 months, 15 years and older and who live/work in this city today? | **\|____\|____\|____\|** | |  |
| SE10 | What is your best guess of the minimum number of women who have willingly sold vaginal or anal sex for money or favors in the past 12 months, 15 years and older and who live/work in this city today? | **\|____\|____\|____\|** | |  |
| SE11 | What is your best guess of the most accurate number of women who have willingly sold vaginal or anal sex for money or favors in the past 12 months, 15 years and older and who live/work in this city today? | **\|____\|____\|____\|** | |  |

| **№** | **Question** | **Code** | **Answer** |
| --- | --- | --- | --- |
| **9.1** | Did you receive a ______________________? | 01=Yes02=No | \|__\|__\| |
| **9.2** | When did you receive the _______________ during last month?*INTERVIEWER: DO YOU BELIEVE THIS PERSON RECEIVED THE UNIQUE OBJECT.* | 01=Yes02=No | \|__\|__\| |

Our questionnaire ends here. If you have question, please ask us!

Thank you for participating in this survey.
