# Supplementary material for: Survival, CD4 T lymphocyte count recovery and immune reconstitution pattern during the first-line combination antiretroviral therapy in patients with HIV-1 infection in Mongolia
Source: PLoS One. 2021 Mar 8;16(3):e0247929. doi: 10.1371/journal.pone.0247929 (PMC7939265; doi:10.1371/journal.pone.0247929)
Supplement: S2 Questionnaire — (DOCX) [file pone.0247929.s002.docx]

**Registration form**

**Men who have sex with men (MSM)**

**Interview date:**

**Day** **Month** **Year**

**Interviewer code**

**Coupon number**

**Interviewee code**

|  |  |  |  |  |  |  |  |
| --- | --- | --- | --- | --- | --- | --- | --- |

**Province/District code**

**Aimag, city code**

01= Ulaanbaatar

02= Darkhan-Uul

03= Dornod

04= Khuvsgul

**Questions to check eligibility for the survey**

**Question 1:** How old are you? **(**Include only men aged between 15 to 49)

**Question 2**: When was the last time you had anal sex with man?

Question 3: Have you been interviewed for this study in the past 3 months? 1 Yes 2 No

**Identifying network**

| **№** | **Questions** | **Code** | **Answers** |
| --- | --- | --- | --- |
| **1** | How many men do you know (who also know you) who are between 15 and 49 who have anal sex with men? | Record the number | \|__\|__\| |
| **2** | How many of them live inUlaanbaatar, Darkhan-Uul, Dornod, Khuvsgul (just use one of these cities depending on where the survey is being conducted) | Record the number (cannot be zero or higher than response to 1) | \|__\|__\| |
| **3** | How many of them have you seen in the past three weeks? | Record the number (cannot be zero or higher than response to 2) | \|__\|__\| |
| **4** | Who gave you this coupon? | 01= Sexual partner  02= Friend  03 = MSM that I know (acquaintance)  04=NGO outreach workers/peer educators(not eligible)  05 = MSM that I do not know well  06. Stranger (not eligible)  07=Other (_____________note) | \|__\|__\| |

**Acceptable condition for the survey**

1= Accepted (Proceed) 2= Not accepted (Do not proceed)

**Confirmation to take interview**

If confirmed, tick √the appropriate box.

If refused, do not proceed.

**Interviewer’s note:**

1. Completed Registration form: 1. Yes 2. No
2. Interviewee’s code on Questionnaire, Registration form,blood collection tube and Laboratory registration are same: 1. Yes 2. No
3. Informed consent has been signed 1. Yes 2. No
4. All questions have been answered: 1. Yes 2. No

Interviewer’s signature: ________________________________ Date: year_____month_____day_____

**Behavioral Survey Questionnaire**

**Men who have sex with men**

**Coupon number:**

**1^st^ section: General information**

| **№** | **Question** | **Code** | **Answer** |
| --- | --- | --- | --- |
| **1.1** | In what year were you born? | *(Record year of birth (4 digits))* | \|__\|__\|__\|__\| |
| **1.2** | What’s your primary/administrative region? | 01 = Ulaanbaatar  02 = Other …………………………………(*note*) |  |
| **1.3** | What is your education Level? | 00= None  01= Primary /1-4grade/  02 =Incomplete secondary /5-8 grade/  03= Complete secondary /9-11 grade/  04= Special Vocational School, College  05= University (means at least bachelor degree)  88 = Don’t want to answer | \|__\|__\| |
| **1.4** | What is your occupational status? (full time work) | 00 = None  01 = Have a full time job (governmental/nongovernmental/private)  02 = Have a part-time job  03 = Student  04 = Other (________________________note)  88 = Don’t want to answer | \|__\|__\| |
| **1.5** | Have you ever been in the army? | 01 = Yes  02 = No | \|__\|__\| |
| **1.6** | Marital Status? | 01= Married  02=Single, never married  03 = Widowed  04 = Divorced | \|__\|__\| |
| **1.7** | Do you currently live with your sex partner? | 01=Yes  02=No | \|__\|__\| |
| **1.8** | If so, is your sex partner male or female? | 01 = Male  02 = Female | \|__\|__\| |

**2^nd^ section: Sexual behavior and attitude**

| **№** | **Question** | **Code** | | | | | | | | | | | **Answer** | |
| --- | --- | --- | --- | --- | --- | --- | --- | --- | --- | --- | --- | --- | --- | --- |
|  | How do you classify your sexual orientation? | 01=Homosexual /Gay  02= Bisexual  03= Heterosexual  04= Other (write) | | | | | | | | | | | \|__\|__\| | |
|  | With which sex do you prefer sexual intercourse? | 01=Males  02=Females  03=Both | | | | | | | | | | | \|__\|__\| | |
|  | At what age did you first have penetrative sexual intercourse? | Age  88 = Don’t want to answer  99 = Don’t remember | | | | | | | | | | | \|__\|__\| | |
|  | Did you use a condom at your first sex? | 01 = Yes  02 = No  99 = Don’t remember | | | | | | | | | | | \|__\|__\| | |
|  | What sex was your first sexual partner? | 01 = Male  02 = Female | | | | | | | | | | | \|__\|__\| | |
|  | Did you want to or did someone force you to do it when you had your first sex? | 01 = I wanted  02 = Someone forced me  03 = Violent matter  04 = I was drunk  05 = Other (________________________note)  88 = Don’t want to answer | | | | | | | | | | | \|__\|__\| | |
|  | **Now I would like to ask some information about male partner** | | | | | | | | | | | | | |
|  | How old were you when you had anal sexual intercourse with a man? | Age  88 = Don’t want to answer  99 = Don’t remember | | | | | | | | | | | \|__\|__\| | |
|  | In what type of anal sex do you mainly engage? | 01 = Active  02 = Passive  03 = Universal  88 = Don’t want to answer | | | | | | | | | | | \|__\|__\| | |
|  | How many times did you have anal sex during the last 7 days? | Numbersof anal sex  88 = Don’t want to answer  99 = Don’t remember | | | | | | | | | | | \|__\|__\| | |
|  | Anal sex in the past 6 months  (ONE RESPONSE FOR COLUMNS A, B AND C) | A.Types of partners | | | | | B.Number of partners | | C. Was a condom used during last sexual intercourse | | | | | |
|  | Partner type | Yes | | No | | | Number | | Yes | | | No | | |
|  | **Steady male sexual partner**(a male person you were/are in a relationship) | 1 | | 2 | | | \|__\|__\| | | 1 | | | 2 | | |
|  | 2.  **Casual male sexual partner** (a man you occasionally have or once had sex without being in a relationship) | 1 | | 2 | | | \|__\|__\| | | 1 | | | 2 | | |
|  | 3.    A man **who paid you** (you sold sex) with money/gifts for anal intercourse | 1 | | 2 | | | \|__\|__\| | | 1 | | | 2 | | |
|  | 4.A man **whom you paid** (you boughit sex) with money/goods for anal sex | 1 | | 2 | | | \|__\|__\| | | 1 | | | 2 | | |
|  | In the last 6 months, how often did you use a condom during anal sex?*(if always used (100%), please move to 2.12)* | | | | | | | | | | | | | |
|  |  | Steady male partner | | | Casual male partner | | | Partner who paid you with money/gifts | | | Partner whom you paid with money/gifts | | | |
|  | Always (100%) | 1 | | | 1 | | | 1 | | | 1 | | | |
|  | Most of the times (75%) | 2 | | | 2 | | | 2 | | | 2 | | | |
|  | About half the time (50%) | 3 | | | 3 | | | 3 | | | 3 | | | |
|  | Sometimes (25%) | 4 | | | 4 | | | 4 | | | 4 | | | |
|  | Rarely (below 10%) | 5 | | | 5 | | | 5 | | | 5 | | | |
|  | Never | 6 | | | 6 | | | 6 | | | 6 | | | |
|  | What were the reasons of you having sex without condom?*There may be multiple choices selected* (for each sexual partner) | | | | | | | | | | | | | |
|  |  | Steady male partner | | | Casual male partner | | | Partner who paid you with money/gifts | | | Partner whom you paid with money/gifts | | | |
|  |  | Yes | No | | Yes | No | | Yes | | No | Yes | | | No |
|  | I do not think sex feels good with a condom | 1 | 2 | | 1 | 2 | | 1 | | 2 | 1 | | | 2 |
|  | Forget to use | 1 | 2 | | 1 | 2 | | 1 | | 2 | 1 | | | 2 |
|  | Didn’t find any | 1 | 2 | | 1 | 2 | | 1 | | 2 | 1 | | | 2 |
|  | There was no time to buy one | 1 | 2 | | 1 | 2 | | 1 | | 2 | 1 | | | 2 |
|  | There was no time to put one on | 1 | 2 | | 1 | 2 | | 1 | | 2 | 1 | | | 2 |
|  | I know the HIV status of my partners | 1 | 2 | | 1 | 2 | | 1 | | 2 | 1 | | | 2 |
|  | Expensive | 1 | 2 | | 1 | 2 | | 1 | | 2 | 1 | | | 2 |
|  | I am embarrassed to buy | 1 | 2 | | 1 | 2 | | 1 | | 2 | 1 | | | 2 |
|  | I don’t trust condoms | 1 | 2 | | 1 | 2 | | 1 | | 2 | 1 | | | 2 |
|  | I use other methods to prevent HIV/STIs | 1 | 2 | | 1 | 2 | | 1 | | 2 | 1 | | | 2 |
|  | My partner does not think sex feels good with a condom | 1 | 2 | | 1 | 2 | | 1 | | 2 | 1 | | | 2 |
|  | I trust my partner | 1 | 2 | | 1 | 2 | | 1 | | 2 | 1 | | | 2 |
|  | Can’t get enough condoms | 1 | 2 | | 1 | 2 | | 1 | | 2 | 1 | | | 2 |
|  | I was drunk | 1 | 2 | | 1 | 2 | | 1 | | 2 | 1 | | | 2 |
|  | Violent matter | 1 | 2 | | 1 | 2 | | 1 | | 2 | 1 | | | 2 |
|  | Other ………………..(note) | 1 | 2 | | 1 | 2 | | 1 | | 2 | 1 | | | 2 |
|  | Can you get condoms every time you need a condom? | 01 = Yes  02 = No  03 = I’ve never had any need  04 =I don’t use condoms (if yes, move to 2.14) | | | | | | | | | | | \|__\|__\| | |
|  | Where do you get condoms when you need one? (DO NOT READ RESPONSE. LET RESPONDENT RESPOND AND THEN ASK,**“ANYTHING ELSE?”**MULTIPLE RESPONSES POSSIBLE. NEED TO CIRCLE **YES** OR **NO** FOR EACH RESPONSE) | | | | | | | | | | | | | |
|  |  | Yes | | | | | | | | | | | No | |
|  | 1. Pharmacy | 1 | | | | | | | | | | | 2 | |
|  | 2. From the sex partner | 1 | | | | | | | | | | | 2 | |
|  | 3. NCCD | 1 | | | | | | | | | | | 2 | |
|  | 4. Zaluus Eruul Mend NGO | 1 | | | | | | | | | | | 2 | |
|  | 5. From the an outreach NGO worker | 1 | | | | | | | | | | | 2 | |
|  | 6. District Health Center | 1 | | | | | | | | | | | 2 | |
|  | 7.Other.............................. (note) | 1 | | | | | | | | | | | 2 | |
|  | Why aren’t you able to buy a condom when you need one? (DO NOT READ RESPONSE. LET RESPONDENT RESPOND AND THEN ASK,**“ANYTHING ELSE?”**MULTIPLE RESPONSES POSSIBLE. NEED TO CIRCLE **YES** OR **NO** FOR EACH RESPONSE) | | | | | | | | | | | | | |
|  | 1.Expensive | Yes | | | | | | | | | | | No | |
|  | 2. Pharmacy is too far | 1 | | | | | | | | | | | 2 | |
|  | 3. Pharmacies are closed when I need a condom | 1 | | | | | | | | | | | 2 | |
|  | 4. Too embarrassed to buy a condom | 1 | | | | | | | | | | | 2 | |
|  | 5. I don’t know where to buy one | 1 | | | | | | | | | | | 2 | |
|  | 6. I dislike carrying a condom | 1 | | | | | | | | | | | 2 | |
|  | 7. Other.............................. (note) | 1 | | | | | | | | | | | 2 | |
|  | Do you know of any organizations that offer free condoms and other services? | 01= Yes  02= No | | | | | | | | | | | \|__\|__\| | |
|  | Have you or your sexual partner ever used a gel or water-based lubricant during anal sex? | 01= Yes  02= No | | | | | | | | | | | \|__\|__\| | |
|  | How often did you use gel or water-based lubricants for anal sex during the last 3 months? | 01= Always 100% (if yes, move to 2.19)  02=Most of the times 75%  03=About half the time 50%  04=Sometimes 25%  05=Never | | | | | | | | | | | \|__\|__\| | |
|  | Why do you not always use gel or water-based lubricants for anal sex? (Multiple choices may be possible) | 01=Do not find it necessary  02=Partner refused  03=Did not have gel,water based lubricants at that time  04=Gel or water based lubricants are not easily available  05=Gel or water based lubricants are expensive  06 = Prefer to use other types of lubricants  07 = Prefer to use other products to improve penetration  88 = Refuse to answer | | | | | | | | | | | \|__\|__\|  \|__\|__\|  \|__\|__\|  \|__\|__\| | |
|  | Which places or persons do you know where you can obtain a gel or water-based lubricant? | 01= Supermarkets  02= Pharmacy  03= Health center  04= Bars and Hotels  05= From a friend  06= NGO  07= Outreach worker  08= Internet  09=Other ………………..(note) | | | | | | | | | | | \|__\|__\|  \|__\|__\|  \|__\|__\|  \|__\|__\| | |
|  | From which place or person did you obtain a gel or water-based lubricant the last time? | 01= Supermarkets  02= Pharmacy  03= Health center  04= Bars and Hotels  05= From a friend  06= NGO  07= Outreach worker  08= Internet  09 = Did not use one  10 =Other ………………..(note) | | | | | | | | | | | \|__\|__\| | |
|  | From which places or persons do you prefer to receive a gel or water-based lubricant? | 01= Supermarkets  02= Pharmacy  03= Health center  04= Bars and Hotels  05= From a friend  06= NGO  07= Outreach worker  08= Internet  09 = Did not use one  10 =Other ………………..(note) | | | | | | | | | | | \|__\|__\| | |

**Now I would like to ask some information about your female sex partners**

| **№** | **Question** | **Code** | **Answer** |
| --- | --- | --- | --- |
|  | Have you ever had penetrative vaginal or anal sex with a female? | 01= Yes  02= No (if no, move to 3.1) |  |
|  | Did you have vaginal or anal sex with a female in the last 12 months? | 01= Yes  02= No (if no, move to 3.1) | \|__\|__\| |
|  | With how many females did you have vaginal or anal sex in the last 12 months? | Number of female partners | \|__\|__\| |
|  | How often did you use a condom during vaginal or anal sex with a female whom you paid for sex in the past 12 months? | 01 = Always (100%)  02 = Most of the time (70-80%)  03 = Sometimes (30-40%)  04 = Rarely (10%)  05 = Never (0%) | \|__\|__\| |
|  | Did you have anal or vaginal sex with a steady female partner in the last 12 months?*(girlfriend, wife)* | 01 = Yes  02 = No | \|__\|__\| |
|  | Did you use condom the last time you had vaginal or anal sex with a steadyfemale partner? | 01 = Yes  02 = No | \|__\|__\| |
|  | Did you have casual sex with a female sexual partner in the last 12 months? | 01= Yes  02= No (if answer is no, please move to 2.20) | \|__\|__\| |
|  | Did you use condom the last time you had casual vaginal or anal sex with a female partner? | 01 = Yes  02 = No | \|__\|__\| |

**Section 3: Knowledge and attitudes of HIV/AIDS and voluntary counselling and testing of HIV/AIDS**

| **№** | **Question** | **Code** | **Answer** |
| --- | --- | --- | --- |
|  | Have you ever heard about HIV/AIDS? | 01=Yes  02=No (if answer is no, please move to 3.6) | \|__\|__\| |
|  | Do you think that having one and faithful sex partner can reduce transmission risk of HIV infection? | 01=Yes  02=No  99 =Don’t know | \|__\|__\| |
|  | Do you think that using condom properly in all sexual intercourses can prevent HIV infection? | 01=Yes  02=No  99 =Don’t know | \|__\|__\| |
|  | Do you think a healthy-looking person can be infected with HIV? | 01=Yes  02=No  99 =Don’t know | \|__\|__\| |
|  | Which type of sex has the highest risk of HIV?  *(Choose one answer only)* | 01 = Vaginal  02 = Anal  03 = Oral  04 = Other (_______________________note)  99 =Don’t know | \|__\|__\| |
|  | What do you consider your risk for contracting HIV to be? | 01=no risk  02=low risk  03=medium risk  04=high risk  05=very high risk  06=I already have HIV | \|__\|__\| |
|  | If you want to be tested for HIV, do you know where to go? | 01=Yes  02=No |  |
|  | Have you ever tested for HIV? | 01=Yes  02=No (if answer is no, please move to 3.14) | \|__\|__\| |
|  | By whose initiativedidyou test for an HIV? | 01=Voluntarily  02=Requiredby employers  03 = Medical worker initiative  04 = Friend initiative  05 = Outreach worker initiative  06 = Other………………………*(note)* | \|__\|__\| |
|  | When was your last HIV tested | 01=6 months  02=6-12 months  03=More than 12 months  04= Don’t want to answer | \|__\|__\| |
|  | Did you receive your test results last time you were tested? | 01=Yes  02=No (if answer is no, please move to 3.14) | \|__\|__\| |
|  | Can you please tell me the result of your last test? | 01= Positive  02=Negative  03= Indeterminate  88 = Don’t want to answer | \|__\|__\| |
|  | If yes, have you received antiretroviral treatment? | 01=Yes  02=No | \|__\|__\| |
|  | Do you agree that attending a regular screening for HIV, STI (every 3-6 months) would help you and your partners to prevent infection? | 01= Yes  02= No  03= Don’t know | \|__\|__\| |
|  | **Which of the following services have you received from an outreach service, drop-in centre or sexual health clinic in the past 3 months?**  (DO NOT READ RESPONSE. LET RESPONDENT RESPOND AND THEN ASK,**“ANYTHING ELSE?”**MULTIPLE RESPONSES POSSIBLE. NEED TO CIRCLE **YES** OR **NO** FOR EACH RESPONSE) | | |
|  |  | Yes | No |
|  | 1.Public events (events, trainings) | 1 | 2 |
|  | 2.Tests for infections that are spread through sexual contact (sexually transmitted infections) | 1 | 2 |
|  | 3.Counselling on condom use and safe sexual practices | 1 | 2 |
|  | 4.Free condoms,lubricants | 1 | 2 |
|  | 5.Other ………………………*(note)* | 1 | 2 |
| **3.16** | Have you ever heard about PEP, or Post-exposure prophylaxis, an emergency HIV treatment? | 01=Yes  02=No (if answer is no, please move to 3.20) | \|__\|__\| |
| **3.17** | How did you hear about PEP? (there may be multiple choices) | 01=Medical personnel  02=NGO  03 = Peer Educators  04 = Friends  05 = Radio  06 = Press  07 = Internet  08 = Other………………………*(note)* | \|__\|__\|  \|__\|__\|  \|__\|__\| |
| **3.18** | Have you ever used PEP? | 01=Yes (if answer is yes, please move to 3.20)  02=No | \|__\|__\| |
| **3.19** | Why have you never used it? | 01=I never felt the need to  02=It was too late, already gone beyond the 72 hours  03 = I feared going to the hospital to ask for it  04 = I was refused PEP  05 = Other………………………*(note)* | \|__\|__\| |
| **3.20** | Have you ever heard about PrEP, or pre-exposure prophylaxis, which can be taken by an HIV negative person before potential HIV exposure? | 01=Yes  02=No (if answer is no, please move to 3.22) | \|__\|__\| |
| **3.21** | How did you hear about PrEP? | 01=Medical personnel  02=NGO  03 = Peer Educators  04 = Friends  05 = Radio  06 = Press  07 = Internet  08 = Other………………………*(note)* | \|__\|__\| |
| **3.22** | *Short explanation on PrEP:* PrEp is when people at very high risk for HIV take HIV medicines daily to lower their chances of getting infected. It is highly effective for preventing HIV if used as prescribed, but it is much less effective when not taken consistently.    If it was available in Mongolia how likely would you have used it? | 01=Very unlikely  02=Unlikely  03 = Not sure  04 = Likely  05 = Very likely  88 = Refuse to answer | \|__\|__\| |
| **3.23** | If PrEP were made available in Mongolia, where would you prefer to access it? | 01=NCCD, AIDS Unit  02=District Health Centre  03 = Private clinics  04 = Pharmacy  05 = NGO  06 = Other………………………*(note)* | \|__\|__\| |

**Section 4: Sexually transmitted infections (STIs)**

| **№** | **Question** | **Code** | **Answer** |
| --- | --- | --- | --- |
|  | Have you had a genital discharge in the last 12 months? | 01 = Yes  02 = No | \|__\|__\| |
|  | Have you had a genital ulcer or sore in the last 12 months? | 01 = Yes  02 = No(if answer is no, please move to 4.1 and 4.2) | \|__\|__\| |
|  | Have you sought treatment for genital discharge, ulcer or sore symptom in the last 12 months? | 01 = Yes  02 = No (if answer is no, please move to 4.5) | \|__\|__\| |
|  | If yes, where did you receive treatment? | 01=NCCD, AIDS Unit  02=District, aimag hospital  03=Private hospital  04 = NGO  05 = Other……………….…*(note)* | \|__\|__\| |
|  | Have you been tested for STI in the last 12 months? | 01=Yes  02=No (if answer is no, please move to 5.1) | \|__\|__\| |
|  | If yes, what were the reasons for getting tested? |  | \|__\|__\| |
|  | If yes, where were you tested? | 01=NCCD, AIDS Unit  02=District, aimag hospital  03=Private hospital  04 = NGO  05 = Other……………….…*(note)* | \|__\|__\| |
|  | What kind of difficulties do you experience when getting tested for STIs other than HIV? | | |
|  |  | Yes | No |
|  | Waited for too long | 1 | 2 |
|  | Work hours of a clinic are unsuitable | 1 | 2 |
|  | Lack of privacy | 1 | 2 |
|  | Negative attitude of the hospital workers (offensive, discriminating) | 1 | 2 |
|  | Hospital environment is uncomfortable (not enough chairs, unhygienic, lack of privacy) | 1 | 2 |
|  | The hospital was too far | 1 | 2 |
|  | The service fee is too high | 1 | 2 |
|  | Other……………….…*(note)* | 1 | 2 |
|  | Were you diagnosed with any of the following in the last 12 months? | | |
|  |  | Yes | No (if no, move to 5.1) |
|  | Syphilis | 1 | 2 |
|  | Gonorrhoea | 1 | 2 |
|  | Chlamydia | 1 | 2 |
|  | If yes, did you receive a treatment? | 01 = Yes  02 = No (if answer is no, please move to 4.12) |  |
|  | If yes, who treated you? | 01= Doctor  02=Pharmacist  03=Self-treated 04= Other……………….…*(note)* | \|__\|__\| |
|  | If not, why did not you seek treatment? *(there may be multiple answers)* | 01 = Symptoms disappeared  02 = Ashamed from other people  03 = Afraid from the doctor  04 = There was no money  05 = Could not find a suitable clinic  06 = Did not know where to approach  07 = No time for approach public clinic  08=Other*………………........……..........(write)* | \|  \|  \| \| --- \| --- \| \|  \|  \| \|  \|  \| \|  \|  \| \|  \|  \| \|  \|  \| \|  \|  \| |

**Section 5: Drug and alcohol use**

| **№** | **Question** | **Code** | **Answer** |
| --- | --- | --- | --- |
|  | Have you consumed alcoholic beverages in the past 12 months? | 01= Yes  02= No (if no, move to5.4) |  |
|  | How often, in the past year, did you use alcoholic beverage? | 01= Once a week  02=More than once a week  03=Once a month  04=Twice a month  05= Once or twice a year | \|__\|__\| |
|  | In the past year, have you used alcoholic beverages before having sex? | 01 = Yes  02 = No | \|__\|__\| |
|  | In the past year, have often did you use alcoholic beverages before having sex? | 01=Always (100%)  02=Usually (75%)  03=Sometimes (50%)  04=Rarely (10%)  88= do not answer | \|__\|__\| |
|  | Have you ever use illicit non-injectable drugs to get high? | 01=Yes  02=No (if answer is no, please move to 6.1) | \|__\|__\| |
|  | If yes, what kind of narcotics have you used? | 1 = Marijuana/Hashish  2 = Cannabis  3 = Ecstasy  4 = Vaporizing substances (glue, acetone, benzine)  5 = Amphetamines  6 = Heroine  07 = Methadone  08 = Cocaine  09=Ice  10=Drugs  11=Mimi  12=Popers  13=  14 = Tranquilizers or sedatives (diazepam, valium, tranqwin, lorazepam, seduxen, tazepam, oxazepam) | \|__\|__\| |
|  | Have you ever injected drugs to get high? | 01=Yes  02=No (if answer is no, please move to 5.12)  99 = Don’t know | \|__\|__\| |
|  | If yes, what kind of injecting drugs have you used? | 01 = Heroine  02 = Cocaine  03 = Morphine  04 = Amphetamines  05 = Prescription drugs (OxyContin, Vicodin, Valium, Alprazolam, Adderall, Ritalin etc.)  06 = Other*………………........……..........(write)* | \|__\|__\| |
|  | If yes, did you share syringe or needle for injection last time you injected drugs? | 01 = Yes  02 = No  99 = Don’t know | \|__\|__\| |

**Section 6:Discrimination and violence**

| **№** | **Questions** | | | **Code** | | | | **Answers** | |
| --- | --- | --- | --- | --- | --- | --- | --- | --- | --- |
|  | Who knows about your sexual orientation?  *“Multiple answers possible".* | | | 01 = Family members  02 = Close friends  03 = Relatives  04 = Colleagues  05 = Only my sexual partner  06 = Other (_______________________note) | | | | \|__\|__\|  \|__\|__\|  \|__\|__\|  \|__\|__\|  \|__\|__\| | |
|  | Have you ever been physically attacked because you have sex with men? | | | 01=Yes, once02=Yes, multiple times03=No04=Refuse answer | | | | \|__\|__\| | |
|  | Have you ever been exposed to violence or abuse from the police because you have sex with men? | | | 01=Yes, once02=Yes, multiple times03=No04=Refuse answer | | | | \|__\|__\| | |
|  | Have you ever been denied health or medical services because you have sex with men? | | | 01=Yes, once02=Yes, multiple times03=No 04 =Refuse answer | | | | \|__\|__\| | |
|  | Have you ever been worried or afraid of getting the following services in the last 12 months? | | | | | | | | |
|  | Service type | Afraid of stigma | | Afraid of someone finding out about my sexual orientation | | Was attacked before/Afraid of violence | | Was arrested by the police before/Afraid of being arrested | |
|  |  | Yes | No | Yes | No | Yes | No | Yes | No |
|  | Health services | 1 | 2 | 1 | 2 | 1 | 2 | 1 | 2 |
|  | HIV screening test | 1 | 2 | 1 | 2 | 1 | 2 | 1 | 2 |
|  | Have you ever been called/detained by law or police for your sexual orientation? | | 01=Yes, once02=Yes, multiple times03=No *04=Refuse answer* | | | | | \|__\|__\| | |
|  | Have you ever wanted an access to a Human Rights organization because of the violation of your right resulting from your sexual orientation? | | 01=Yes, once02=Yes, multiple times03=No *04=Refuse answer* | | | | | \|__\|__\| | |
|  | Have you ever been a victim of a sexual violence? | | 01=Yes, once02=Yes, multiple times03=No *04=Refuse answer* | | | | | \|__\|__\| | |

**Size Estimation.** Questions for RDSfor Multiplier Population Size Estimation

| **NO.** | **QUESTIONS AND FILTERS** | **CODING CATEGORIES** | | **SKIP** |
| --- | --- | --- | --- | --- |
| **Unique object multiplier** | |  | |  |
| SE1 | Did you receive a medicine storage in the recent past? (Prompt if not willing to say/doesn’t remember) | Yes 1  No 2 | | 🡺SE5 |
| SE2 | Which is the medicine storage you received? (show picutre of different key rings) | Yes 1  No 2 | | 🡺SE5 |
| SE3 | How many medicine storage did you receive? | **\|____\|____\|** | |  |
| SE4 | When did you receive this medicine storage?  Must be around (enter distribution dates) 2019 | _ _/ _ _ _ _ (mm/yyyy) | |  |
| SE5 | Interviewer: Did participant receive a medicine storage? | Yes 1  No 2 | |  |
| **Service multiplier** | |  | |  |
| SE6 | Between April4 and June 30, did you get an HIV test at NGO named Zaluus Eruul Mend? | Yes 1  No 2 | |  |
| SE7 | Between April 4 and June 30, did you get free condoms at NGO named Zaluus Eruul Mend? | Yes 1  No 2 | |  |
|  | Between January 1 and June 30, did you enroll behavioural change communication program at NGO named Zaluus Eruul Mend? | Yes 1  No 2 | |  |
| **App service multiplier** | |  | |  |
| SE8 | From August 2019 to September 2019 (one month before survey) did you use any of the following apps for the purposes of meeting other men for sex? How many different times during the month did you log on to the app? | APP  FACEBOOK  More | TIME LOGGING IN  ___ |  |
|  |  |  |  |  |
| **Wisdom of the crowds for MSM** | |  |  |  |
| SE9 | What is your best guess of the maximum number of men who have anal sex in the past 12 months, 15 years and older and who live/work in this city today? | **\|____\|____\|____\|** | |  |
| SE10 | What is your best guess of the minimum number of men who have anal sex in the past 12 months, 15 years and older and who live/work in this city today? | **\|____\|____\|____\|** | |  |
| SE11 | What is your best guess of the most accurate number of men who have anal sexin the past 12 months, 15 years and older and who live/work in this city today? | **\|____\|____\|____\|** | |  |

Our questionnaire ends here. If you have question, please ask from us!

Thank you for participating survey.
